# So many ways to naturally kill a cancer cell

**DOI:** 10.1186/s12915-021-01092-3

**Published:** 2021-07-30

**Authors:** Timothy J. Mitchison

**Affiliations:** Department Systems Biology, Harvard Medical School, 200, Longwood Ave, Boston, MA 02115 USA

## Abstract

Natural killer (NK) cells participate in cancer immunosurveillance and cancer immunotherapy. Live cell imaging of cancer cells targeted by NK cells, published today in BMC Biology by Zhu et al., reveals a remarkable diversity of programmed cell death pathways induced in individual cells. Pathway choice depends on the state of the target cell actin cytoskeleton and a novel death pathway, granzyme-induced necroptosis, could be of broad importance in cancer immunotherapy.

## NK cells in cancer

NK cells are cytotoxic lymphocytes of the innate immune system. They lack the highly variable repertoire of somatically generated receptors for non-self-antigens present on T-lymphocytes of the adaptive immune system, but can recognize features of altered-self and kill virus-infected and cancer cells [1]. NK cells interrogate potential targets using multiple activating and inhibitory cell surface receptors, integrating over multiple cues before firing their cytotoxic arsenal. One important activating cue is the loss of expression of major histocompatibility complex (MHC) molecules on the target cell surface. MHC molecules present foreign or altered-self peptides to the adaptive immune system, and viruses often block MHC expression to prevent recognition of infected cells by T cells, while cancer cells may be selected for MHC downregulation to escape immunosurveillance. The ability of NK cells to recognize and kill cancer cells makes them useful in cancer therapy to remove cancer cells from patient bone marrow ex vivo, as an in vivo cell-based treatment, and as a component of the response to immune checkpoint inhibitors (ICIs) [2, 3]. The receptors that allow NK cells to distinguish cancer vs non-cancer cells have been extensively characterized, as have cancer resistance mechanisms that depend on altered expression of cell surface ligands for these receptors. In contrast, the cell death mechanisms that NK cells trigger in diverse cancer cells, on which Zhu et al. [4] shed new light, have been less studied.

## NK cell killing mechanisms

NK cells share target cell killing mechanisms with cytotoxic T cells [5]. Killing is mediated by secreted and cell-surface proteins that induce programmed cell death pathways in the target cell, which has to actively cooperate. Target killing occurs by two cellular pathways, cytotoxic granule-dependent and granule-independent. Cytotoxic granules are specialized, acidic secretory vesicles that contain perforin and granzymes which are secreted into the immunological synapse between a cytotoxic lymphocyte and its target. Perforin forms pores in the target cell membrane which allow granzymes to access the target cell cytoplasm [6]. NK and T cells express multiple granzymes that kill targets in different ways, perhaps to make it difficult for viruses and cancer to evolve resistance to killing. The best characterized is granzyme B, a protease that cleaves and activates caspase 3 to induce target cell apoptosis [6]. Granule-independent killing is mediated by secretion and/or cell surface expression of death receptor ligands such as FAS ligand and TRAIL [5]. These bind to death receptors on the target cell and induce apoptosis via caspase 8 activation. They can also trigger non-apoptotic death pathways such as necroptosis and pyroptosis [7].

## Diversity in cancer cell responses

**Fig. 1:**
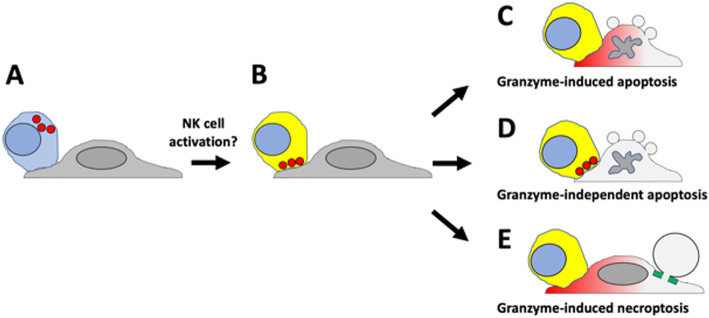
Key methods and results from Zhu et al. [4]. **a** Primary NK cells (blue) were co-cultured with cancer cells (gray) on a microscope stage. The blue color indicates a NK cell that is not yet activated. The red circles are cytotoxic granules which are not yet polarized towards the target cell. Granules were imaged using a dye that accumulates in acidic compartments. **b** If the NK cell detected an activating balance of ligands on the target cell surface, it committed to killing the target, illustrated by the yellow color. Cytotoxic granules then move towards the target cell on microtubules prior to directed secretion into the immunological synapse. **c** Granzyme-induced apoptosis is the canonical cell-killing pathway for both NK and T cells. It is typically induced when granzyme B cleaves and activates caspase 3 in the target cell. The red gradient indicates uptake of granzymes by the target cell. Granzyme B activity was monitored using a fluorescent biosensor. Apoptosis was monitored using a reporter of mitochondrial outer membrane permeabilization (MOMP). **d** Granzyme-independent apoptosis is a well-characterized cell killing pathway that is triggered by death receptor ligands expressed by the NK cell. It was scored by apoptosis that occurred in the absence of granzyme B activation and did not require granule secretion. **e** Granzyme-dependent necroptosis is a novel cell killing pathway. It was scored by large, sparse blebs on the target cell, lack of MOMP, dependence on cytotoxic granule secretion, granzyme B activity, and expression of RIP1, RIP3, and MLKL. The green bar indicates the assembly of MLKL-dependent pores at the base of a bleb, which is the execution step in necroptosis. Necroptosis blebs are distinguished from apoptosis blebs by their larger size, much smaller number per cell and lack of rapid inflation-retraction dynamics.

Cancer cells exhibit huge diversity in their response to NK and T cells. This may explain divergent responses to therapy with engineered NK and T cells as well as immune checkpoint inhibitors (ICIs) that activate T and NK cells in situ [2, 3]. ICIs are now the first-line treatment for many cancers, and cell-based therapies using engineered T and NK cells are increasing in importance. To enhance the effectiveness of these therapies, it is important to understand variability in cancer cell responses. Zhu et al. [4] investigated how several cancer cell lines respond to primary NK cells using live-cell microscopy and fluorescence biosensors (Fig. 1). This approach allowed separate measurement of the extent of target cell killing and the cell death pathway chosen by individual target cells. The rate and extent of killing were largely determined by the expression of known cell surface ligands for NK cell receptors, which confirms the importance of these receptors in controlling whether NK cells fire their cytotoxic arsenal [1]. The originality of Zhu et al.’s study [4] comes from their characterization of alternative cell death pathways downstream of NK cell activation on a cell-by-cell basis. Using fluorescence biosensors, they defined three death pathways (Fig. 1). Examples of each pathway, plus the no-death outcome, were observed in individual cells in all four cancer cell lines studied. However, their relative prevalence varied greatly, with one or two different outcomes predominant in each line.

## Granzyme-induced necroptosis

The discovery of this novel death pathway, which predominated in MCF7 cells, is probably the most far-reaching contribution of this new research [4]. Necroptosis is a programmed cell death pathway triggered by RIPK1 kinase where the execution step is the insertion of MLKL-dependent pores into the plasma membrane [8]. Necroptosis was discovered more recently than apoptosis and is involved in the pathology of many diseases. Zhu et al. [4] observed that MCF7 target cells developed large, sparse blebs a few minutes after NK cell contact, which they recognized as a cytological signature of necroptosis (Fig. 1e). They went on to demonstrate dependence on necroptosis pathway proteins RIPK1, RIPK3, and MLKL. NK cell-induced necroptosis in MCF7 cells depended on cytotoxic granule secretion and was inhibited by a granzyme B inhibitor. Different granzymes kill target cells by diverse pathways [6], and NK cells were previously reported to induce necroptosis via death receptor ligands [7], but this is the first report, to my knowledge, of activation of necroptosis by granzyme B. Granzyme B is best known for activating caspase 3 to trigger apoptosis, but it has many other substrates [6]. MCF7 cells lack caspase 3, which might explain why necroptosis is triggered instead. Zhu et al.’s finding suggests necroptosis may be an effector pathway for cytotoxic lymphocytes more generally, especially in apoptosis-resistant cancer cells.

Cancer cell killing by NK and T cells is thought to play a major role in immunosurveillance and response to therapy with ICIs and engineered T cells [2, 3]. Previous research assumed that apoptosis, triggered by granzymes or death receptor ligands, was the main killing mechanism, implying that immunosurveillance leads to the selection of apoptosis-resistant cancer clones. Zhu et al.’s study [4], along with other recent work implicating necroptosis in cancer therapy [9], suggests that necroptosis resistance may also be selected during cancer progression. If this hypothesis is validated, the rescue of necroptosis by pharmacology, or engineering therapeutic lymphocytes to trigger it more efficiently, will be important new directions. Necroptosis inhibitors are under development for inflammatory, neurological, and fibrotic diseases [8]. NK and T cell killing may contribute to the pathology of such diseases, which adds another dimension to the potential therapeutic relevance of granzyme-induced necroptosis.

## Cortical actin in death pathway choice

The cortical actin cytoskeleton powers cell migration and modulates the signaling activity of cell surface receptors. Zhu et al. observed correlations between cortical actin dynamics and choice of death pathway and went on to show that modulating actomyosin had a strong effect on death pathway choice, though not on the fraction of cells that were killed [4]. This is not the first-time actin has been connected to programmed cell death, for example, actomyosin-dependent generation of many small blebs is a cytological signature of apoptosis [10]. It is the first report, to my knowledge, of a role of actin dynamics in modulating the choice of death pathway. The precise effect of actomyosin modulators on death pathways differed between cell lines, so untangling the biology will be complex. Cell culture on stiff 2D surfaces poorly models the mechanical microenvironment of tumors and has a huge effect on cortical actin, so studies in more realistic models, such as tumor organoids, will be required.

## Perspective

How NK and T cells kill cancer cells is a profoundly cell biological question, yet it is rarely addressed using cell biology’s most powerful tool, live imaging with fluorescent reporters. Zhu et al.’s study demonstrates the value of live imaging for pathway discovery and quantification. An important limitation was its reliance on 2D culture on stiff substrates, which makes imaging easy, but is a poor model for the mechanical microenvironment in tumors. Given the limited number of cell lines examined and artificial modulation of cortical actin by 2D culture, it is not possible to extrapolate directly from the Zhu et al. study to human tumors. The huge diversity of human cancers makes it likely that all the death pathways observed by Zhu et al. [4], and perhaps others, are relevant in particular patients. The trick will be predicting which predominates in a given patient and learning how to enhance cancer cell killing by NK and T cells in that patient. In closing, I note that most research in cancer therapeutics assumes that apoptosis is the main route to tumor regression. Perhaps necroptosis has been underestimated and enhancing it could improve pharmacologic as well as cell-based therapies.

## Data Availability

Not applicable
